# The Safety and Efficacy of a Proprietary Bioactive Fatty Acids Extract From Saw Palmetto ( *Serenoa repens* ) for Promoting Hair Growth and Reducing Hair Loss in Adults With Self‐Perceived Thinning Hair: 90‐Day Results

**DOI:** 10.1111/jocd.70585

**Published:** 2025-11-30

**Authors:** Glynis Ablon

**Affiliations:** ^1^ Ablon Skin Institute and Research Center Manhattan Beach California USA

**Keywords:** alopecia, clinical trial, hair shedding, male and female pattern hair loss, saw palmetto, *Serenoa repens*, thinning hair

## Abstract

**Background:**

Hair loss is a global concern for both men and women.

**Aims:**

This study assessed the efficacy and safety of a proprietary extract of bioactive fatty acids from saw palmetto for treating self‐perceived thinning hair in healthy adult male and female subjects.

**Methods:**

In this 6‐month, randomized, double‐blind, placebo‐controlled study, subjects were randomized to receive active treatment (*n* = 40) or placebo (*n* = 20). Half of the enrolled subjects were female. Subjects took one oral dose of their assigned treatment daily. Assessments were performed at baseline, Day 56 and Day 90. Preliminary 90‐day results are presented here.

**Patients/Methods:**

The active group showed significant improvement in terminal hair count from baseline in anterior (*p* < 0.0007) and posterior (*p* < 0.0005) areas. Compared to placebo, the improvement was also significant in the anterior (*p* < 0.05) and posterior (*p* < 0.03) areas. Anterior vellus hair count improved significantly versus baseline (*p* < 0.0005) and placebo (*p* < 0.01). Posterior vellus hair count showed significant within‐group growth (*p* < 0.05). Total hair count was significantly greater than baseline for anterior (*p* < 0.001) and posterior (*p* < 0.0005) and significantly greater for anterior (*p* < 0.005) and posterior (*p* < 0.05) compared to placebo. Total terminal hair counts improved 7‐fold, and total hair count improved 12‐fold compared to placebo. A significant reduction in hair shedding was observed in the active group versus baseline (*p* < 0.05). No meaningful improvements were observed in the placebo group, and no adverse events were reported.

**Conclusion:**

These preliminary results indicate the daily administration of a proprietary saw palmetto ( *Serenoa repens* ) extract safely and effectively promotes hair growth in men and women with self‐perceived thinning hair.

**Trial Registration:**

Clinicaltrials.gov identifier: NCT06920758

## 1 Introduction

The problem of hair loss is a global concern for both men and women [1]. While the two most common causes of hair loss are androgenetic alopecia and alopecia areata [2, 3], hair loss is a cause of significant global psychological distress in both men and women, regardless of the underlying cause [4, 5]. Recent studies have reported male pattern hair loss alone affects approximately 80% of males by the age of 80 [6] and female pattern hair loss affects 40% of women by age 50 [7].

Pharmacotherapy for hair loss is limited. For male pattern hair loss, initial treatment can include oral or topical minoxidil to which oral finasteride may be added [8]. Similarly for female pattern hair loss, initial drug therapy can include oral or topical minoxidil to which oral finasteride may be added in postmenopausal women [8]. Until recently, there were no other FDA‐approved treatments for hair loss in the United States. In 2023, the FDA announced approval of ritlecitinib (Litfulo, Pfizer Inc.) for the treatment of severe alopecia areata [9].

Saw palmetto, or *Serenoa repens*, is a plant from the Arecaceae family. Oil extracted from its berries contains 85%–90% fatty acids, including capric, caprylic, lauric and myristic acids, and *β*‐sitosterol [10]. Extracts of *Serenoa repens* are nonselective competitive inhibitors of 5‐alpha reductase Types I and II. This is a similar mechanism of action as finasteride but with a superior safety profile [11]. By blocking the enzyme that converts testosterone to its active metabolite dihydrotestosterone [12], *Serenoa repens* promotes follicle health and encourages hair growth. The efficacy of topical *Serenoa repens* for treating hair loss such as male androgenetic alopecia has previously been demonstrated [13, 14]. Similar *Serenoa repens* extracts have demonstrated benefits for the treatment of benign prostatic hyperplasia [15].

Following an analysis of available research data and clinical evidence, a novel, proprietary bioactive fatty acids extract from saw palmetto ( *Serenoa repens* ) has been developed for maintaining hair health and treating hair loss (SEREVELLE, formerly USPlus DERM, Valensa International; Eustis, FL). The product is formulated as concentrated bioactive free fatty acids that benefit hair health, the hair growth cycle and other desirable hair characteristics.

A recent 12 weeks study assessed the effectiveness of topical application of *Serenoa repens* extract and bioactive free fatty acids (SEREVELLE) for treating hair loss in healthy adults (*N* = 22) [16]. Assessment at 12 weeks revealed significant improvements in hair loss and increased hair density in both male and female subjects which began as early as 4 weeks with an overall high degree of subject satisfaction.

Based on these promising results, this 6‐month randomized, double‐blind, placebo‐controlled study was conducted to evaluate the safety and efficacy of an oral SEREVELLE formulation for promoting hair growth and reducing hair loss in adults with self‐perceived thinning hair. The results of an interim 90‐day evaluation are presented here.

## 2 Methods

### 2.1 Study Subjects

Study participants were healthy male and female subjects, 25–65 years old, with any Fitzpatrick skin type who were seeking treatment for self‐perceived hair thinning. Male subjects were required to have frontal and/or vertex patterns I, II, IIA, III, III vertex, and IV based on the investigator‐rated Norwood classification scale [17]. Female subjects were required to have investigator‐rated scores of I‐1, I‐2 and I‐3 based on the Savin pictorial scale [18]. All subjects expressed their willingness to follow all study requirements including brief physical examinations, digital imaging, maintaining their current hair length, style and color, attending all study visits, and adhering to their current diet, medications, and exercise routines for the duration of the study.

Reasons for exclusion from the study included hair transplants or hair extensions, a stressful incident within the last 6 months (e.g., death in the family, miscarriage), or any of the following within the previous 3 months: initiation of hormonal birth control or hormone replacement therapy, use of low‐level laser therapy or other light therapy to treat thinning hair, use of minoxidil or finasteride or other hair or scalp treatments, use of prescription drugs known to affect the hair growth cycle (e.g., cyproterone acetate, spironolactone, or 5‐alpha‐reductase inhibitors) or antiandrogen therapies (i.e., flutamide, cyproterone acetate, or progesterone). Other reasons for exclusion included other hair loss disorders (e.g., alopecia areata, scarring alopecia, telogen effluvium), uncontrolled systemic disorders (e.g., diabetes mellitus, hypertension, hyperthyroidism), active hepatitis, immune deficiency, HIV or autoimmune disease, a scalp condition that may place the subject at risk or interfere with clinical evaluations (e.g., seborrheic dermatitis, psoriasis, atopic dermatitis), pregnancy, lactation, or planned pregnancy during the course of the study, or simultaneous participation in another clinical study.

### 2.2 Investigational Product

The study treatment was a proprietary concentration of bioactive fatty acids from saw palmetto, important for hair structure and characteristics, and with potent activity against 5*α*‐reductase‐1 [19]. It was provided as an oral formulation containing 160 mg of concentrated proprietary bioactive fatty acids extract from saw palmetto ( *Serenoa repens* ) in a soft capsule comprised of bovine gelatin, glycerin, and water (SEREVELLE, Valensa International, Eustis, FL). The placebo consisted of identical‐appearing capsules containing palm oil. To maintain stability, the investigational product was maintained at room temperature (59°F–86°F). Subjects were to take one dose of their assigned treatment daily.

### 2.3 Study Endpoints

The three primary efficacy endpoints of this 90‐day interim analysis were changes in baseline anterior and posterior terminal hair counts, changes in baseline anterior and posterior vellus hair counts, and changes in baseline anterior and posterior total hair counts compared to placebo.

Secondary endpoints were changes in terminal‐to‐vellus ratio, mean number of hairs per follicular unit, mean hair width, number of follicular units per cm^2^, mean interfollicular distance, investigator hair growth global improvement scale, investigator hair quality global improvement scale scores, change in hair shedding pull test scores, hair self‐assessment questionnaire scores, hair thinning quality of life questionnaire scores, and hair product subject satisfaction questionnaire.

The safety endpoint was the nature, severity and frequency of treatment‐related adverse events, and tolerability of the investigational product.

### 2.4 Study Procedures

This study was planned as a 90‐day, double‐blind, randomized, placebo‐controlled study with a 6‐month follow‐up to evaluate the efficacy of the active treatment to promote hair growth in adult men and women. After an initial screening period to determine eligibility, subjects were randomized to receive active treatment (*n* = 40) or placebo (*n* = 20) for 3 months. After visit 1 (Day 0), subjects returned to the study site at visit 2 (Day 90). Subjects received a phone call on Day 56 to complete a compliance check and subject questionnaires. Two‐dimensional digital images of the entire head/hair region were obtained on visits 1 and 2 (IntelliStudio System. Canfield Scientific; Parsippany, NJ). These images were used to aid in grading assessments.

### 2.5 Trichoscopy Imaging

Two 1 cm^2^ target sites were selected on the scalp of each subject for digital imaging for performing hair growth assessments. One site was located along the frontalis bone where the frontal and lateral hairlines meet. A 3‐point location was noted on each subject based on measurements obtained from the medial canthus, lateral canthus, and preauricular skin pit to the target site area. Where these three points met, the target site was identified with a black skin marker. The second site was in the posterior scalp transitional zone. This was chosen from the top of the head at least 1 cm anterior to the edge of the vertex thinning/balding area of the scalp and located using the hair check locating strip. After identifying the selected area, the template was used to mark a midline area in the transition zone, not in the thinning or balding spot. The midpoint within this area was marked with a black marker. The investigator ensured that the target sites were representative of a transitional zone between active hair thinning and healthier hair. Trichoscopy imaging of the two target site areas was performed on visits 1 and 2 to measure hair counts per cm^2^ (terminal, vellus and total), terminal‐to‐vellus ratio, mean hairs per follicular unit, mean hair width, follicular units per cm^2^ and interfollicular mean distances (HairMetrix, Canfield Scientific, Parsippany, NJ).

In addition to the combined cohort of male and female subjects, a subgroup analysis was performed on nonchildbearing women who were in a premenopausal or menopausal stage of reproduction. This subgroup was analyzed separately due to the well‐documented influence of hormonal fluctuations during reproductive years on hair growth and shedding patterns. This analysis was performed to enable a clearer understanding of the efficacy of SEREVELLE in this population. This was a within‐group analysis as an equivalent group of placebo‐treated subjects was unavailable.

### 2.6 Hair Shedding Pull Test

The hair pull test was performed by the investigator on visits 1 and 2. In this test, gentle traction was applied to a group of approximately 60 hairs from the proximal to distal end, in the vertex area, both parietal areas and the occipital area of the scalp. The grasp is made with the thumb, index finger and middle finger, ensuring consistent pressure with each pull. If ≥ 6 hairs are released with each pull, the test is considered positive [20]. For accurate results, subjects were instructed not to shampoo or wash their hair for 24 h prior to the test.

### 2.7 Other Assessments

The investigator completed global hair assessments using a 7‐point Likert scale corresponding to descriptions that best matched the current global hair growth and current hair quality for each subject on Day 56 and Day 90. Assessment grading was based upon the investigator's appraisal of hair brittleness, dryness, texture, shine, scalp coverage and overall appearance using global imaging obtained during the study on Day 90 visit compared to the visit 1 baseline images together with the investigator's clinical judgment from in‐person observations.

Subjects completed a self‐assessment questionnaire consisting of questions related to their opinion of changes in their hair at the Day 56 phone call and at visit 2 which were compared to the visit 1 baseline responses. Subjects also completed a hair thinning quality of life questionnaire consisting of questions related to their opinion of how their thinning hair affected their daily life during the Day 56 phone call and visit 2 which were compared to visit 1 baseline responses.

### 2.8 Statistical Analysis

Each of the efficacy endpoints was analyzed separately. Sample size calculations were based on the intended primary efficacy endpoint using a Student's *t*‐test analysis with a significance level of 0.5 and power of 80%, anticipating a mean change in terminal hair count of +10 for the active group and +5 for the placebo group and a standard deviation of ±5.3. This resulted in a sample size of 18 subjects per group which was increased to 20 in consideration of a potential withdrawal/loss to follow‐up rate of 10%. Given the 2:1 active: placebo enrollment ratio, the sample size was established as 40 subjects randomized to the active group and 20 subjects randomized to the placebo group. A 2‐tailed Student's *t*‐test for two independent samples was used to assess the statistical significance of the mean difference in baseline (Day 0) hair counts between active treatment and placebo groups, a 2‐tailed Student's *t*‐test for two correlated samples was used to assess the significance of the mean difference in baseline hair counts within each active treatment group, and a 2‐tailed Student's *t*‐test for two independent samples was used to assess the significance of the mean difference in baseline hair counts between active treatment groups.

### 2.9 Ethics

Each subject provided written informed consent prior to participating in any study‐related activities. This study protocol and related documents were approved by a commercial institutional review board (Allendale IRB, Old Lyme, CT). The conduct of this study followed all applicable guidelines for the protection of human subjects for research as outlined in the United States FDA 21 CFR Part 50, in accordance with the accepted standards for good clinical practices (GCPs). Each subject also provided written HIPAA and Photography Release Forms.

## 3 Results

Sixty subjects were randomized to the active (*n* = 40) and placebo (*n* = 20) groups with male and female subjects equally divided in each group. The demographics and clinical characteristics of enrolled subjects are summarized in Table 1. There were no significant between group differences in these parameters. Similarly, there were no significant between group differences in physical examination assessments including body weight, height, blood pressure, heart rate, respiratory rate, or body temperature.

**TABLE 1:** Subject demographics and clinical characteristics.

	Active	Placebo
	*n* = 40	*n* = 20
Mean age, years (SD)	50.6 (9.6)	48.9 (10.9)
Median age, years (min, max)	55.0 (28, 63)	50.5 (29, 63)
Race, *n* (%)	*n* = 40	*n* = 20
White	36 (90)	14 (70)
Black	1 (2.5)	1 (5)
Asian	1 (2.5)	2 (10)
American Indian/Alaskan Native	—	2 (10)
Native Hawaiian/Pacific Islander	—	1 (5)
Mixed race	2 (5)	—
Ethnicity, *n* (%)	*n* = 40	*n* = 20
Hispanic/Latino	32 (80)	15 (75)
Non‐Hispanic/Latins	8 (20)	5 (25)
Fitzpatrick skin type	*n* = 40	*n* = 20
Type I	6 (15)	5 (25)
Type II	14 (35)	4 (20)
Type III	12 (30)	5 (25)
Type IV	6 (15)	5 (25)
Type V	2 (5)	—
Type VI	—	1 (5)
Norwood classification scale (male subjects), *n* (%)	*n* = 20	*n* = 10
II	1 (5)	1 (10)
III	8 (40)	7 (70)
III Vertex	11 (55)	2 (20)
Savin pictorial scale (female subjects), *n* (%)	*n* = 20	*n* = 10
1–2	15 (75)	7 (70)
1–3	5 (25)	3 (30)

*Note:* There were no significant between‐group differences.

### 3.1 Primary Endpoints, Day 90

Preliminary 90‐day results indicated the mean (SD) baseline anterior terminal hair count increased by 11.3 (19.4) for the active treatment group (*p* < 0.001) versus a mean decrease of 2.7 (14.2) for the placebo group (*p* = NS). The mean baseline posterior terminal hair counts increased by 12.1 (18.7) for the active treatment group (*p* < 0.001) versus 0.5 (18.6) for the placebo group (*p* = NS). The difference between groups was significant (*p* < 0.03). Total terminal hair count (anterior + posterior) increased by 23.4 (27) for the active treatment group (*p* < 0.001) versus baseline and total terminal hair retention and growth was 7.8 × larger than placebo (*p* < 0.004).

The mean baseline anterior vellus hair count increased by 4.4 (9.2) for the active treatment group (*p* < 0.004) versus a mean decrease of 2.7 (10.7) for the placebo group (*p* = NS). The difference between groups was significant (*p* < 0.015). There were no significant mean changes in posterior vellus hair counts for either group.

**FIGURE 1:**
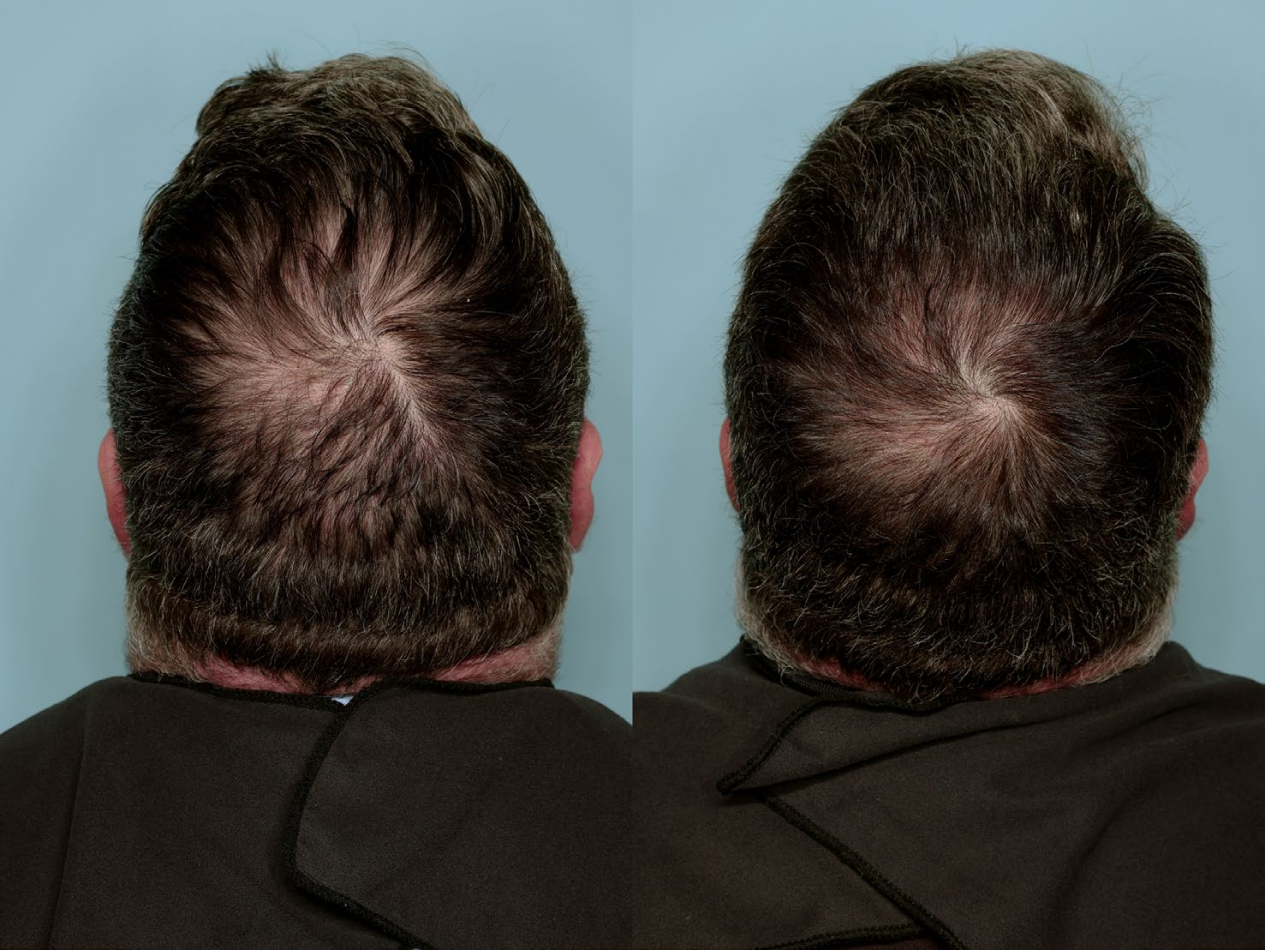
The increase in baseline hair thickness (left) is apparent in this male subject after 90 days of daily administration of an oral formulation containing 160 mg of concentrated proprietary bioactive fatty acids extract from saw palmetto ( *Serenoa repens* ) (right).

**FIGURE 2:**
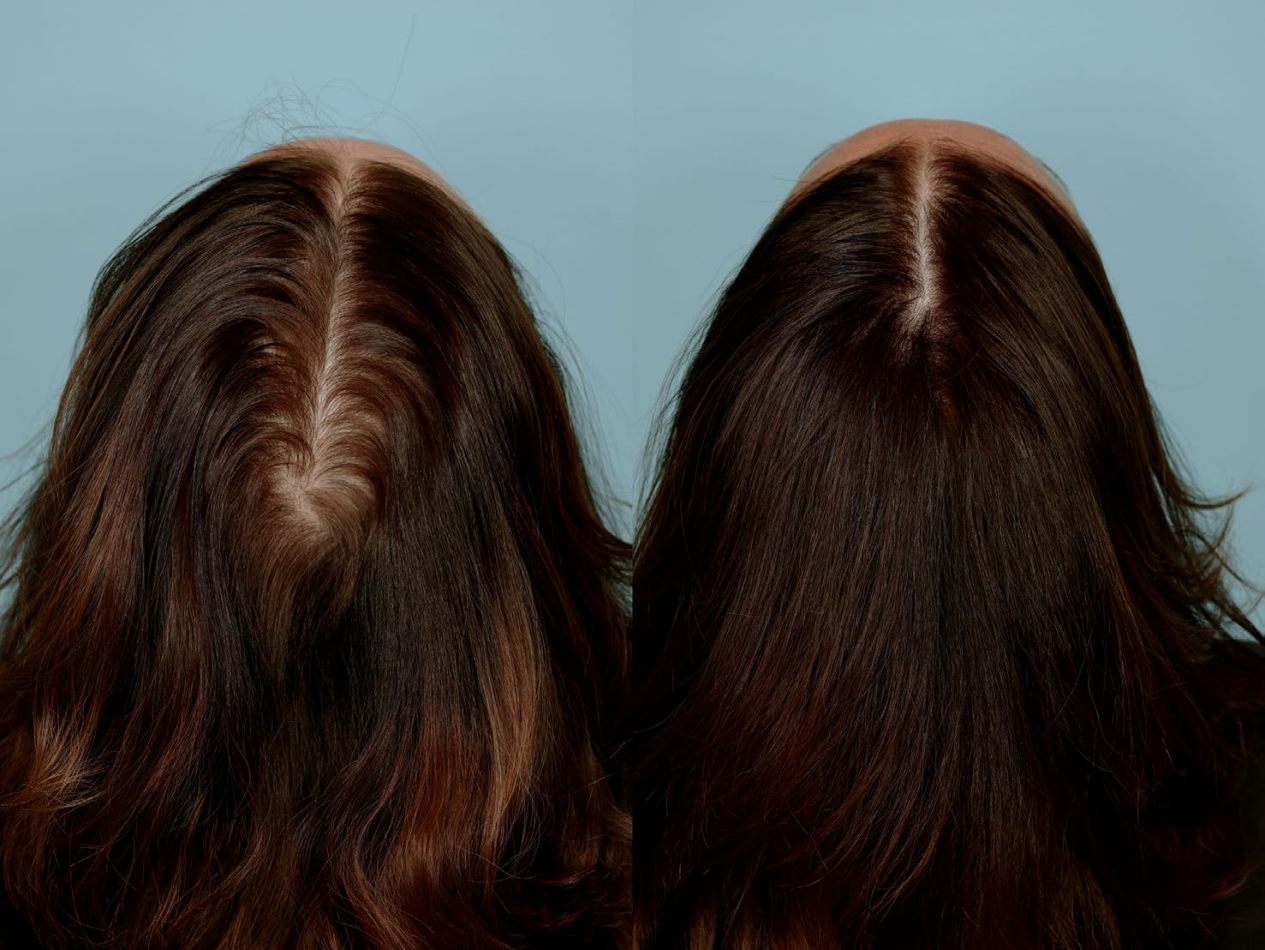
The increase in baseline hair thickness (left) is apparent in this female subject after 90 days of daily administration of an oral formulation containing 160 mg of concentrated proprietary bioactive fatty acids extract from saw palmetto ( *Serenoa repens* ) (right).

The mean baseline anterior total hair count increased by 15.7 (18.5) for the active treatment group (*p* < 0.001) versus a mean decrease of 0.1 (19.8) for the placebo group (*p* = NS). The mean baseline posterior total hair count increased by 13.1 (21.4) for the active treatment group (*p* < 0.0005) versus a mean increase of 2.3 (18.5) for the placebo group (*p* = NS). Total hair count (anterior + posterior) increased by 28.8 (28.3) for the active treatment group (*p* < 0.001) versus baseline and total terminal hair count retention and growth was 12.8 × larger than placebo (*p* < 0.004). These results are summarized in Table 2. The improvement in hair thickness is apparent in representative male (Figure 1) and female subjects (Figure 2).

**TABLE 2:** Primary endpoint. Hair counts by treatment group, Day 0 and Day 90.

	Active (*n* = 40)			Placebo (*n* = 20)		
	Day 0	Day 90	Change	Day 0	Day 90	Change
**Anterior terminal hair counts**						
Mean (SD)	132.9 (29.2)	144.2 (32.4)	+11.3 (19.4)^a,b^	131.8 (30.7)	134.4 (28.9)	+2.7 (14.2)
Median (min, max)	134.5 (60, 202)	145.0 (70, 206)	10.5 (70, 206)	137.5 (85, 199)	135.5 (85, 210)	2.0 (−28, 34)
^a^ *p* = 0.00068 versus baseline, 2‐tailed Student's *t*‐tests for correlated samples. ^b^ *p* < 0.05.						
**Posterior terminal hair counts**						
Mean (SD)	167.1 (32.9)	179.2 (34.0)	+12.1 (18.7)^a,b^	175.2 (34.6)	175.6 (31.7)	+0.6 (18.6)
Median (min, max)	168 (100, 233)	183 (105, 258)	12 (−20, 41)	175 (123, 246)	172 (117, 232)	3 (−39, 31)
^a^ *p* < 0.0005 versus baseline, 2‐tailed Student's *t*‐test for correlated samples. ^b^ *p* < 0.05 versus placebo, 2‐tailed Student's *t*‐test for independent samples.						
**Anterior vellus hair counts**						
Mean (SD)	10.8 (9.5)	15.2 (12.6)	+4.4 (9.2)^a,b^	14.9 (10.9)	12.2 (11.4)	−2.7 (10.7)
Median (min, max)	8 (0, 37)	11 (0, 50)	3.5 (−11, 28)	14 (0, 35)	8.5 (0, 41)	−1.5 (−32, 11)
^a^ *p* < 0.0005 versus baseline, 2‐tailed Student's *t*‐test for correlated samples. ^b^ *p* < 0.01 versus placebo, 2‐tailed Student's *t*‐test for independent samples.						
**Posterior vellus hair counts**						
Mean (SD)	4.8 (6.6)	5.8 (6.9)	+1.0 (8.0)	3.5 (3.1)	5.4 (4.7)	+1.9 (4.2)
Median (min, max)	1 (0, 29)	3 (0, 31)	0 (−22, 20)	3 (0, 11)	4.5 (0, 14)	0 (−6, 10)
**Anterior total hair counts**						
Mean (SD)	143.7 (28.0)	159.4 (30.7)	+15.7 (18.5)^a,b^	146.7 (33.3)	146.6 (27.4)	−0.1 (19.8)
Median (min, max)	146.5 (66, 206)	155.5 (97, 219)	13.5 (−31, 52)	142 (98, 222)	139.5 (101, 213)	−1 (−36, 39)
^a^ *p =* NS versus baseline, 2‐tailed Student's *t*‐test for correlated samples. ^b^ *p* < 0.005 versus placebo, 2‐tailed Student's *t*‐test for independent samples.						
**Posterior total hair counts**						
Mean (SD)	171.9 (35.4)	185.0 (36.0)	+13.10 (21.4)^a,b^	178.7 (33.3)	181.0 (30.2)	+2.3 (18.5)
Median (min, max)	171 (100, 246)	190 (105, 272)	11 (−42, 58)	178 (130, 249)	174.5 (127, 232)	3 (−31, 39)
^a^ *p* = 0.0004 versus baseline, 2‐tailed Student's *t*‐test for correlated samples. ^b^ *p* < 0.05 versus placebo, 2‐tailed Student's *t*‐test for independent samples.						

### 3.2 Subgroup Analysis of Nonchildbearing‐Age Women

In the subgroup of nonchildbearing‐age women (*n* = 15), treatment with the active product led to a significant improvement in anterior terminal hair counts, with a mean increase of 7.7% hairs over 90 days (*p* < 0.05). The anterior total hairs also showed a substantial rise in total hair counts, with a mean gain of 15.9 hairs or a 11.2% increase (*p* < 0.005). The mean anterior vellus hair counts increased by 5.9 hairs or 43.2% (Table 3). In the secondary endpoints, the most prominent results were in reduction in hair shedding by 70.7% (*p* < 0.05), a 6.6% increase in follicular units/cm^2^ (*p* < 0.01), and anterior sum of hair width per cm^2^ increased by 13.4% (*p* < 0.05).

**TABLE 3:** Primary endpoint. Hair counts for active treatment group, nonchildbearing‐age women, *n* = 15.

	Day 0	Day 90	Change	Day 0	Day 90	Change
	**Anterior terminal hair counts**			**Posterior terminal hair counts**		
Mean (SD)	128.6 (23.76)	138.6 (29.45)	+10 (15.27)^a^	185.87 (23.98)	191.8 (27.49)	+5.93 (19.45)
Median (min, max)	128 (82, 169)	142 (90, 206)	8 (−15, 37)	194 (142, 220)	193 (146, 258)	6 (−20, 39)
^a^ *p* < 0.05 versus baseline, 2‐tailed Student's *t*‐tests for correlated samples.						
	**Anterior vellus hair counts**			**Posterior vellus hair counts**		
Mean (SD)	13.6 (9.88)	19.47 (15.59)	+5.87 (11.46)	5.47 (8.88)	6.00 (5.98)	+0.53 (9.28)
Median (min, max)	12 (0, 37)	20 (0, 50)	6 (−11, 28)	0 (0, 29)	6 (0, 15)	0 (−22, 15)
	**Anterior total hair counts**			**Posterior total hair counts**		
Mean (SD)	142.2 (23.15)	158.07 (28.15)	+15.87 (16.77)^a^	191.33 (28.95)	197.8 (29.5)	+6.47 (22.47)
Median (min, max)	145 (96, 178)	155 (110, 206)	13 (−11, 52)	194 (142, 238)	196 (146, 272)	10 (−42, 39)
^a^ *p* < 0.005 versus baseline, 2‐tailed Student's *t*‐test for correlated samples.						

### 3.3 Secondary Endpoints (Day 90)

Among subjects in the active treatment group, there was a significant decrease in mean (SD) baseline anterior interfollicular distance of 0.03 (0.08) mm (*p* < 0.05), an increase in the mean number anterior follicular units per cm^2^ of 7.5 (12.6) (*p* < 0.05), and mean posterior follicular units per cm^2^ of 6.4 (17.02) (*p* < 0.001), an increase in mean baseline anterior sum of hair widths per cm^2^ of 990.1 (1807.9) (*p* < 0.005) and mean baseline posterior sum of hair widths per cm^2^ of 769.8 (1304.5) (*p* < 0.001) which was significantly greater than 6.0 (1478.4) for the placebo group (*p* < 0.05). There were no significant improvements in any secondary endpoints among subjects in the placebo group.

For the active treatment group, there was a significant decrease in the mean number of baseline total number of hairs shed in the hair pull test of 0.4 (1.1) (*p* < 0.05). This change was significantly greater than the mean decrease of 0.2 (1.3) in the placebo group (*p* < 0.05). Although there was a decrease in the mean number of shed hairs in each individual scalp area, they were not significant. There were no significant decreases among placebo‐treated subjects (Table 4).

**TABLE 4:** Hair shedding pull test.

	Active (*n* = 40)			Placebo (*n* = 20)		
	Day 0	Day 90	Change	Day 0	Day 90	Change
**Total number of hairs (summed across all regions), *n* (%)**						
Mean (SD)	1.2 (1.0)	0.8 (0.9)	−0.4 (1.1)^a^	1.1 (1.3) 1.0 (0.9)	1.0 (0.9)	−0.2 (1.3)
Median (min, max)	1 (0, 3)	1 (0, 3)	0 (−3, 2)	1 (0, 4)	1 (0, 4)	0 (−3, 4)
^a^ *p* < 0.05 versus baseline, 2‐tailed Student's *t*‐test for correlated samples						
**Number of hairs: vertex, *n* (%)**						
Mean (SD)	0.5 (0.6)	0.3 (0.6)	−0.2 (0.7)	0.8 (0.8)	0.4 (0.6)	−0.4 (0.9)
Median (min, max)	0 (0, 2)	0 (0, 2)	0 (−2, 1)	1 (0, 3)	0 (0, 2)	0 (−2, 2)
**Number of hair: left parietal, *n* (%)**						
Mean (SD)	0.2 (0.5)	0.1 (0.2)	−0.1 (0.5)	0.1 (0.3)	0.1 (0.2)	−0.1 (0.2)
Median (min, max)	0 (0, 2)	0 (0, 1)	0 (−1, 1)	0 (0, 1)	0 (0, 1)	0 (−1, 0)
**Number of hairs: right parietal, *n* (%)**						
Mean (SD)	0.2 (0.4)	0.1 (0.3)	−0.1 (0.6)	0.2 (0.4)	0.3 (0.4)	0.1 (0.6)
Median (min, max)	0 (0, 1)	0 (0, 1)	0 (−1, 1)	0 (0, 1)	0 (0, 1)	0 (−1, 0)
**Number of hairs: occipital, *n* (%)**						
Mean (SD)	0.4 (0.7)	0.3 (0.5)	−0.1 (0.7)	0.3 (0.7)	0.3 (0.4)	−0.1 (0.9)
Median (min, max)	0 (0, 3)	0 (0, 2)	0 (−2, 1)	0 (0, 3)	0 (0, 1)	0 (−3, 1)

The investigator ratings of change in hair quality and hair growth are summarized in Table 5. The greater improvement in rating for subjects in the active treatment group was readily apparent. The investigator ratings of change in hair quality by category for the active treatment group were overall improved (*n* = 32, 80%) (*p* < 0.05). The investigator ratings of change in hair growth by category for the active treatment group were increased (*n* = 24, 60%) (*p* < 0.05).

**TABLE 5:** Investigator ratings of change, Day 90.

Rating *n* (%)	Active (*n* = 40)	Placebo (*n* = 20)
**Hair quality**		
Greatly improved	1 (2.5)	—
Moderately improved	10 (25)	4 (20)
Slightly improved	21 (52.5)	6 (30)
No change	3 (7.5)	7 (35)
Slightly worsened	5 (12.5)	3 (15)
Moderately worsened	—	—
Greatly worsened	—	—
**Hair growth**		
Greatly increased	—	—
Moderately increased	9 (23)	2 (10)
Slightly increased	15 (38)	4 (20)
No change	9 (23)	9 (45)
Slightly decreased	7 (18)	5 (25)
Moderately decreased	—	—
Greatly decreased	—	—

Among subjects in the active treatment group, significant ratings were achieved for overall hair volume (*p* < 0.05), and hair quality (*p* < 0.005) for changes in hair and skin characteristics questionnaire on Days 56 and 90.

Subjects responded to the hair thinning quality of life questionnaire on Days 0, 56 and 90. Possible responses to each question were 4, Very Much; 3, A Lot; 2, A Little; 1, Not at All; or 0, Not Relevant.

1. I am embarrassed by my thinning hair.
2. My thinning hair impacts my self‐esteem.
3. My thinning hair makes me feel self‐conscious.
4. My thinning hair makes me less outgoing than I would like to be.
5. My thinning hair makes me feel unattractive.
6. My thinning hair makes me feel stressed.
7. My thinning hair makes me feel anxious.

A trend analysis revealed little change in ratings for either group across the three time points. In general, the “Not at All” responses in the active treatment group trended slightly upwards from Day 0 to Day 56 and stabilized at dDy 90. Among subjects in the active treatment group, there was a significant increase in the number of baseline “Not at All” responses to question 6 (*p* < 0.05) and question 7 (*p* < 0.05).

### 3.4 Safety Endpoint (Day 90)

There were no reported local or systemic adverse events considered probably or possibly related to the investigational product.

## 4 Discussion

Hair loss is a global concern affecting both men and women, yet despite the prevalence, there are few therapeutic treatment options. Topical minoxidil formulations are available over‐the‐counter for both men and women but may not be effective or tolerated for all patients. Adverse events in women include unwanted facial hair growth [21]. Oral finasteride is available for treating hair loss in men but is not specifically indicated for women due to teratogenicity concerns [22]. Finasteride is not without some safety issues. Common adverse events included erectile dysfunction, decreased libido, depression, suicidal ideation, psychotic disturbances and attention disorders [23]. Consequently, new and more effective treatments are always being sought.

Among the 40 subjects treated with SEREVELLE in the current study, significant increases in mean baseline hair counts were evident in the active treatment group at the Day 90 assessment for anterior terminal hairs (+11.33, *p* < 0.01), posterior terminal hairs (+12.1, *p* < 0.0005), anterior vellus hairs (+4.4, *p* < 0.005), anterior total hairs (+15.7, *p* < 0.001), and posterior total hairs (+13.1, *p* < 0.0005), indicating increased hair density and volume. Increased vellus hairs suggest hair regrowth and decreased miniaturization often found in hair loss. In contrast, changes observed at Day 90 for subjects in the placebo group were negative or negligible and not statistically significant. In addition, the difference in the mean between‐group change in baseline measures at Day 90 was significantly greater among subjects in the Active treatment group for posterior terminal hairs (+11.7, *p* < 0.05), anterior vellus hairs (+7.0, *p* < 0.01), and anterior total hairs (+15.8, *p* < 0.005). Importantly, these results indicate the efficacy of SEREVELLE, a proprietary bioactive fatty acid extract from *Serenoa repens* for treating thinning hair in male and female subjects. Overall, the secondary endpoints supported the trend revealed by the primary outcomes analysis, with some Day 90 measures demonstrating significant improvement but only for the active supplement group. For example, there was a significant decrease in mean baseline of the anterior interfollicular distance (*p* < 0.05), an increase in the mean number of anterior follicular units per cm^2^ (*p* < 0.05) and an increase in mean baseline anterior sum of hair widths per cm^2^ (*p* < 0.001). Also, there was an increase in mean baseline anterior sum of hair widths per cm^2^ (*p* < 0.005) and the posterior sum of hair widths per cm^2^ (*p* < 0.001), all indicating better coverage with increased density and thicker, fuller hair. The active treatment group also had significantly less shedding with a decrease in the mean number of hairs shed compared to baseline (*p* < 0.05), indicating less hair loss.

The results of the preliminary subgroup analysis suggest that the product may be particularly effective among nonchildbearing‐age women, with the most pronounced benefits seen in the anterior region of the scalp, a cosmetically important area for female hair appearance. The anterior total hairs showed a substantial rise in total hair counts, suggesting a combined enhancement of terminal and vellus hair density. The increase in mean anterior vellus hair counts suggests early follicular activation or possible reversal of hair miniaturization.

Subjects in the active treatment group rated their treatment outcomes very highly. Those subjects gave positive ratings for 21 of 24 hair and skin characteristics on the hair self‐assessment questionnaire at Day 90 (87.5%) compared with 15 of 24 questions (62.5%) for the Placebo group (*p* < 0.05). For specific hair characteristics, there were significant improvements among subjects in the active treatment group at Day 90 for overall hair volume (*p* < 0.05) and hair quality (*p* < 0.005).

Extracts of *Serenoa repens* are known inhibitors of 5‐alpha‐reductase, blocking the formation of dihydrotestosterone [24]. Several studies have demonstrated the effectiveness of *Serenoa repens* formulations for treating hair loss but only assessed the treatment of male androgenetic alopecia [25, 26]. Two studies assessing the use of *Serenoa repens* included female subjects; however, both used oral formulations and combined *Serenoa repens* with other botanicals [14, 27]. One recent topical study demonstrated the efficacy of SEREVELLE for treating hair loss in healthy adults including female subjects using the same formulation used in the present study [16]. Although there were only seven female subjects, they achieved a significant 25% decrease in hair loss. Thus, the results of the current study which was evenly comprised of men and women provide supporting evidence of the effectiveness of SEREVELLE, a proprietary bioactive fatty acids extract from saw palmetto ( *Serenoa repens* ) for treating hair loss in this underserved population.

*Serenoa repens* appears to have an excellent safety record. Most adverse events associated with the use of oral *Serenoa repens* for alopecia are mild and most commonly gastrointestinal in nature, including nausea, constipation, and diarrhea [11]. In the present study, there were no adverse events reported with SEREVELLE extract.

## 5 Conclusion

The preliminary 90‐day results of this randomized double‐blind, placebo‐controlled study indicate the oral supplementation of a proprietary bioactive fatty acids extract from saw palmetto ( *Serenoa repens* ) safely and effectively promotes hair health and growth, while reducing hair shedding in men and women with self‐perceived thinning hair.

## Disclosure

Photo Consent Statement: Each subject also provided written HIPAA and Photography Release Forms.

## Ethics Statement

The author confirms that the ethical policies of the journal, as noted on the journal's author guidelines page, have been adhered to and the appropriate ethical review committee approval has been received.

## Conflicts of Interest

The author declares no conflicts of interest.

## Data Availability

The data that support the findings of this study are available from the corresponding author upon reasonable request.
